# Supplementary material for: Prevalence of hypertension and possible risk factors of hypertension unawareness among individuals aged 30–75 years from two Panamanian provinces: Results from population-based cross-sectional studies, 2010 and 2019
Source: PLoS One. 2022 Nov 28;17(11):e0276222. doi: 10.1371/journal.pone.0276222 (PMC9704556; doi:10.1371/journal.pone.0276222)
Supplement: S1 Table — (PDF) [file pone.0276222.s001.pdf]

S1 Table.
Definition of exposure variables based on the study year.

Exposure variables	Studies	Study questionnaire	Assessment	Missing value N (%)	Observations
**Demographic and socioeconomic variables**					
Age	PREFREC 2010<br>ENSPA 2019	*How old are you now?*<br>Fill the blank.<br>*Age?*<br>Fill the blank.	Continuous and categorical variable ("<50 years" or "≥50 years")	-<br>-	-
Sex	PREFREC 2010<br>ENSPA 2019	*Sex?*<br>Options: "men" or "women".<br>*Sex of interviewee.*<br>Options: "men" or "women".	Dichotomic variable "men" or "women"	-<br>-	Based on the phenotypic characteristic.
Ethnicity	PREFREC 2010<br>ENSPA 2019	*To which socio-cultural milieu do you belong?*<br>Options: "Afro-Panamanian", "Mestizo", "Asian", "Indigenous", "Caucasian", or "others"	Categorical variable "Afro-Panamanian", "Caucasian", "Mestizo", "Indigenous", or "others (including Asians)".	-<br>-	Asians were grouped with "others" due to small numbers.
Region	PREFREC 2010<br>ENSPA 2019	Based on INEC definitions [1] using the last Panamanian Census 2010.<br>Options: "urban", "rural", or "indigenous".	Dichotomic variable "urban" "non-urban"	-<br>-	Indigenous and rural regions were grouped into "non-urban" due to small numbers.
Education	PREFREC 2010<br>ENSPA 2019	*How many years of education?*<br>Options: "never attended", "primary education (one to six years)", "secondary education (seven to twelve years)", "technical (thirteen years)", "university (fourteen to eighteen years)", or "post-graduate (nineteen years)".<br>*What is the highest level of education you've achieved?*<br>Options: "no formal education", "special education", "incomplete primary education", "complete primary education", "incomplete secondary education", "complete secondary education", "technical", "non-university higher education", "completed university", "incomplete university", "post-graduate", or "other".	Categorical variable "no/primary education" "secondary education" "higher education"	men: 3611 (1.4)<br>women: 5064 (1.0)<br>men: 7975 (1.2)<br>women: 6001 (0.9)<br>("others" was set to missing)	Special education (ENSPA) was grouped with "no/primary education" and technical education was grouped with "secondary education".<br>University and post-graduate were grouped into "higher education".
Monthly Family Income	PREFREC 2010<br>ENSPA 2019	*What is the monthly family income?*<br>Options: "<250 PAB", "250–300 PAB", "301–600 PAB", "601–999 PAB", "1000–1200 PAB", ">1200 PAB", or "Don't know".<br>*Could you give me an estimate of the monthly family income?*<br>Options: "Doesn't have", "1–74 PAB", "75–99 PAB", "100–124 PAB", "125–149 PAB", "150–174 PAB", "175–199 PAB", "200–249 PAB", "250–299 PAB", "300–399 PAB", "400–499 PAB", "500–599 PAB", "600–699 PAB", "700–799 PAB", "800–899 PAB", "900–999 PAB", "1000–1499 PAB", "1500–1999 PAB", ">2000 PAB", or "Don't know".	Categorical variable "less than 250 PAB" "250–999 PAB" "≥ 1000 PAB".	men: 4291 (1.7)<br>women: 32268 (6.1)<br>("don't know" was set to missing)<br>men: 30969 (4.6)<br>women: 34992 (5.2)<br>("don't know" was set to missing)	Although the PAB is the official currency of Panama, the United States dollar (USD) is also a *de facto* currency. The exchange rate between PAB and USD is set at parity, according to Panamanian law 84/1904. [2]
**Established risk factors for hypertension**					
	PREFREC 2010	*Have you ever smoked a tobacco product in your life?*<br>Options: "yes", or "no".	Categorical variable	men: 921 (0.4)	We did not consider assessing both the consumption of electronic nicotine

Tobacco consumption	ENSPA 2019	*Have you used cigarettes or other smoked tobacco products in the previous 30 days?*<br>Options: "yes" or "no".<br><br>*At any time in your life, how often do you use smoked tobacco?*<br>Options: "daily", "not daily", or "never".<br>*How often did you use smoked tobacco in the previous 30 days?*<br>Options: "daily", "not daily", or "never".	"current smoker"<br>(Smoked 30 days before answering the survey)<br>"ex-smoker"<br>(Smoked more than 30 days prior to replying to the survey)<br>"non-smokers"	women: 480 (0.1)<br><br>men: 1 (0.0)<br>women: 156 (0.0)	delivery systems and non-smoke tobacco products because they were not asked in the PREFREC survey. Furthermore, results from the Global Adult Tobacco Survey in 2013 showed that the prevalence of non-smoked tobacco products consumption in Panama was 0.8%. [3]
Physical inactivity	PREFREC 2010<br><br>ENSPA 2019	Indicator: used the amount of time in minutes per week participants reported doing certain physical activities.<br><br>Indicator: used the Global Physical Activity Questionnaire [4] to classify individuals based on their METs.	Dichotomic variable<br>"physical inactivity"<br>(less than 150 minutes of certain physical activity per week or less than 600 MET-minutes per week)	-<br><br>men: 135212 (20.3)<br>women: 124222 (18.3)	Physical inactivity was defined as not meeting current WHO recommendations on physical activity for health. [4]
Body Mass Index	PREFREC 2010<br><br>ENSPA 2010	Weight: Two measurements were taken using a calibrated instrument. If the 1st and 2nd measurement had a difference of >0.5 kg, a third measurement was performed.<br>Height: Two measurements were taken. If the 1st and 2nd measurement had a difference of >0.5 cm, a third measurement was performed.<br>The average of the measurements was considered the participant's weight and height.	Categorical variable<br>"underweight" (<18.5 kg/m^2)<br>"normal weight" (18.5 to 24.9 kg/m^2)<br>"overweight" (25 to 29.9 kg/m^2)<br>"obesity" (≥30 kg/m^2)	Men: 1940 (0.8)<br>Women: 10251 (1.9)<br><br>Men: 87786 (13.2)<br>Women: 76261 (11.3)	Calculated by dividing the participant's weight in kilograms by the square of their height in meters squared.<br><br>Classification was based on WHO criteria. [5]
Family history of hypertension	PREFREC 2010<br><br>ENSPA 2019	*Have any of your family members (parents, grandparents, aunts, uncles, siblings) been diagnosed with hypertension?*<br>Options: "yes", "no", or "don't know ".<br>*Have any of your family members (parents, grandparents, aunts, uncles, siblings) been diagnosed with hypertension?*<br>Options: "yes" or "no".	Dichotomic variable<br>"yes" or "no"	Men: 16480 (6.5)<br>Women: 16919 (3.2)<br>("Don't know" was set to missing)<br><br>-	-
Self-reported medical history of diabetes	PREFREC 2010<br><br>ENSPA 2019	*Have you been told by a physician that you have diabetes?*<br>Options: "yes" or "no".<br><br>*Has a physician diagnosed you with type 1 diabetes?* or *has a physician diagnosed you with type 2 diabetes?*<br>Options: "yes" or "no".	Dichotomic variable<br>"yes" or "no"	-<br><br>-	-
**BP assessment in the year before study enrollment**	PREFREC 2010<br><br>ENSPA 2019	*When was the last time a health professional checked your blood pressure?*<br>Options: "in the previous year", "1–5 years", "more than 5 years", or "never".<br>*Have you had your blood pressure checked in the previous year?*<br>Options: "yes" or "no".<br>Additional question: *How many health check-ups do you have each year?*<br>Options: "none", "1–2", or ">3".	Dichotomic variable<br>"yes" or "no"	-<br><br>-	-

N=weighted study population. INEC= Spanish language for "National Institute of Statistics and Census". PAB=Panamanian Balboa. METs= Metabolic Equivalents.

Reference:

1. National Institute of Statistics and Census. Glossary of terms [cited 28 June 2021]. Available from: https://www.inec.gob.pa/glosario/Default.aspx?ORDEN=L&ID_IDIOMA=1
2. Torres JE. Population, economy, and society in Panama: a contribution to the critique of Panamanian historiography. Editorial Universitaria "Carlos Manuel Gasteazoro"; 1999; p. 307.
3. Global Adult Tobacco Survey. Panama 2013. Fact sheet [cited 9 July 2021]. Available from: https://www.who.int/tobacco/surveillance/survey/gats/pan_factsheet2013.pdf
4. World Health Organization. Global Physical Activity Surveillance [cited 9 July 2021]. Available from: https://www.who.int/ncds/surveillance/steps/GPAQ_EN.pdf
5. Harvard T.H. Chan website. Obesity Prevention Source: Why Use BMI? [cited 27 July 2021]. Available from: https://www.hsph.harvard.edu/obesity-prevention-source/obesity-definition/obesity-definition-full-story/
