# Supplementary material for: Prevalence of hypertension and possible risk factors of hypertension unawareness among individuals aged 30–75 years from two Panamanian provinces: Results from population-based cross-sectional studies, 2010 and 2019
Source: PLoS One. 2022 Nov 28;17(11):e0276222. doi: 10.1371/journal.pone.0276222 (PMC9704556; doi:10.1371/journal.pone.0276222)
Supplement: S2 Table — Odds ratio (OR) and 95% confidence intervals (CIs). (PDF) [file pone.0276222.s002.pdf]

S2 Table.

Crude and adjusted logistic regression analysis of hypertension prevalence (SBP/DBP ≥140/90 mmHg detected during the study and/or self-reported medical history of hypertension) in participants aged between 30 and 75 years in the provinces of Panama and Colon using merged data from the PREFREC (2010) and ENSPA (2019) studies. Odds ratio (OR) and 95% confidence intervals (CIs).

	**Crude analysis OR (95% CI)**	**Adjusted analysis OR (95% CI)**	
		Model A	Model B
**Study Year**			
PREFREC (2010) study (reference)	1 (ref)	1 (ref)	1 (ref)
ENSPA (2019) study	**1.16 (0.98–1.37)**	0.82 (0.64–1.04)	0.79 (0.62–1.02)
**Demographic Factors**			
Sex			
Women (reference)	1 (ref)	1 (ref)	1 (ref)
Men	0.84 (0.71–1.00)	**0.74 (0.60–0.92)**	**0.70 (0.56–0.89)**
Age group			
≥50 years (reference)	1 (ref)	1 (ref)	1 (ref)
<50 years	**3.76 (3.18–4.45)**	**3.60 (2.95–4.38)**	**3.40 (2.76–4.19)**
Ethnicity			
Caucasian (reference)	1 (ref)	1 (ref)	1 (ref)
Afro-Panamanian	1.11 (0.85–1.44)	1.01 (0.74–1.38)	1.00 (0.73–1.37)
Mestizo	1.14 (0.91–1.42)	1.17 (0.90–1.53)	1.15 (0.88–1.52)
Indigenous	**3.59 (2.22–5.80)**	**2.09 (1.18–3.72)**	**2.49 (1.36–4.55)**
Others (including Asians)	1.36 (0.81–2.27)	0.99 (0.56–1.73)	0.87 (0.52–1.47)
**Established risk factors for hypertension**			
Tabaco consumption			
Non-smoker (reference)	1 (ref)	1 (ref)	1 (ref)
Ex-smoker	**0.71 (0.54–0.91)**	1.09 (0.80–1.49)	1.16 (0.84–1.58)
Current smoker	0.94 (0.65–1.38)	0.83 (0.50–1.38)	0.84 (0.49–1.44)
Body mass index categories[1]			
Underweight	1.87 (0.84–4.13)	1.41 (0.59–3.36)	1.38 (0.56–3.40)
Normal weight (reference)	1 (ref)	1 (ref)	1 (ref)
Overweight	0.81 (0.65–1.01)	**0.70 (0.55–0.90)**	**0.66 (0.51–0.86)**
Obesity	**0.50 (0.40–0.62)**	**0.41 (0.31–0.54)**	**0.38 (0.29–0.50)**
Physical inactivity			
No (reference)	1 (ref)	1 (ref)	1 (ref)
Yes	1.03 (0.86–1.23)	0.94 (0.75–1.19)	0.95 (0.74–1.21)
Family history of hypertension			
No (reference)	1 (ref)	1 (ref)	1 (ref)
Yes	**0.38 (0.32–0.45)**	**0.41 (0.33–0.50)**	**0.40 (0.33–0.50)**
Self-reported medical history of diabetes			
No (reference)	1 (ref)	1 (ref)	1 (ref)
Yes	**0.28 (0.21–0.39)**	**0.41 (0.29–0.59)**	**0.41 (0.29–0.59)**
**BP assessment in the year before study enrollment**			
No (reference)	1 (ref)	1 (ref)	1 (ref)
Yes	**0.42 (0.36–0.50)**	**0.54 (0.44–0.67)**	**0.53 (0.43–0.66)**
**Socioeconomic Factors**			
Region			
Urban (reference)	1 (ref)		1 (ref)
Non-urban	**1.02 (0.86–1.20)**		0.93 (0.73–1.17)
Education			
Higher education (reference)	1 (ref)		1 (ref)
Secondary education	0.95 (0.77–1.18)		1.05 (0.80–1.37)
No/primary education	**0.55 (0.44–0.70)**		**0.71 (0.52–0.98)**
Monthly Family Income			
≥1,000 PAB (reference)	1 (ref)		1 (ref)
250–999 PAB	1.07 (0.85–1.37)		0.85 (0.64–1.13)
<250 PAB	0.87 (0.67–1.13)		0.72 (0.50–1.03)

SBP=systolic blood pressure. DBP=diastolic blood pressure. OR=odds ratio. CIs=confidence intervals. BP=blood pressure. BMI=body mass index. PAB=Panamanian Balboa.

Model A= adjusted by study year, sex, age group, ethnicity, BMI categories, physical inactivity, family history of hypertension, self-reported medical history of diabetes, tobacco consumption, and BP assessment in the year before study enrollment.

Model B= further adjusted by region, education, and monthly family income.

[1]According to World Health Organization (WHO).
