# Supplementary material for: Prevalence of hypertension and possible risk factors of hypertension unawareness among individuals aged 30–75 years from two Panamanian provinces: Results from population-based cross-sectional studies, 2010 and 2019
Source: PLoS One. 2022 Nov 28;17(11):e0276222. doi: 10.1371/journal.pone.0276222 (PMC9704556; doi:10.1371/journal.pone.0276222)
Supplement: S4 Table — Odds ratio (OR) and 95% confidence intervals (CIs). (PDF) [file pone.0276222.s004.pdf]

**S4 Table.**

**Crude and adjusted logistic regression analysis of hypertension unawareness (SBP/DBP ≥140/90 mmHg without a self-reported medical history of hypertension) in hypertensive participants aged between 30 and 75 years in the PREFREC study in the provinces of Panama and Colon. Odds ratio (OR) and 95% confidence intervals (CIs).**

| **Demographic Factors** | **Crude analysis OR (95% CI)** | **Adjusted analysis OR (95% CI)** Model 1 | Model 2 | Model 3 | Model 4 |
|---|---|---|---|---|---|
| Sex | | | | | |
| Women (reference) | | | | | |
| Men | **2.49 (1.60 – 3.89)** | **2.30 (1.33 – 3.96)** | **2.32 (1.30 – 4.13)** | **2.31 (1.29 – 4.13)** | **2.31 (1.29 – 4.12)** |
| Age group | | | | | |
| ≥50 years (reference) | | | | | |
| <50 years | **1.69 (1.07 – 2.65)** | **1.68 (1.01 – 2.80)** | 1.61 (0.95 – 2.73) | 1.61 (0.95 – 2.72) | **1.84 (1.06 – 3.17)** |
| Ethnicity | | | | | |
| Caucasian (reference) | | | | | |
| Afro-Panamanian | 1.34 (0.65 – 2.79) | 1.07 (0.47 – 2.43) | 1.05 (0.46 – 2.41) | 1.05 (0.46 – 2.41) | 1.17 (0.51 – 2.72) |
| Mestizo | 0.96 (0.52 – 1.80) | 0.94 (0.47 – 1.87) | 0.96 (0.47 – 1.97) | 0.96 (0.47 – 1.96) | 1.04 (0.50 – 2.16) |
| Indigenous | 0.91 (0.23 – 3.55) | 1.26 (0.30 – 5.22) | 1.22 (0.29 – 5.15) | 1.20 (0.29 – 5.04) | 1.47 (0.37 – 5.92) |
| Others (including Asians) | 2.40 (0.59 – 9.77) | 0.95 (0.23 – 3.88) | 0.58 (0.12 – 2.93) | 0.59 (0.12 – 2.96) | 0.74 (0.14 – 3.98) |
| **Risk factors for hypertension** | | | | | |
| Tabaco consumption | | | | | |
| Non-smoker (reference) | | | | | |
| Ex-smoker | 1.28 (0.78 – 2.10) | 0.90 (0.50 – 1.62) | 0.81 (0.44 – 1.48) | 0.81 (0.44 – 1.48) | 0.81 (0.45 – 1.47) |
| Current smoker | 1.64 (0.66 – 4.10) | 0.56 (0.16 – 1.95) | 0.56 (0.16 – 1.98) | 0.56 (0.16 – 1.99) | 0.56 (0.16 – 1.97) |
| Body mass index[1] | | | | | |
| Healthy weight (reference) | | | | | |
| Underweight | 3.38 (0.88 – 12.98) | 2.62 (0.52 – 13.11) | 2.40 (0.48 – 11.89) | 2.36 (0.47 – 11.81) | 2.36 (0.46 – 12.01) |
| Overweight | 0.70 (0.41 – 1.18) | 0.64 (0.37 – 1.09) | 0.60 (0.35 – 1.04) | 0.60 (0.35 – 1.04) | 0.63 (0.36 – 1.10) |
| Obesity | **0.45 (0.25 – 0.80)** | **0.45 (0.24 – 0.84)** | **0.45 (0.24 – 0.84)** | **0.45 (0.24 – 0.85)** | **0.48 (0.25 – 0.91)** |
| Physical inactivity | | | | | |
| No (reference) | | | | | |
| Yes | **0.54 (0.29 – 0.99)** | **0.47 (0.25 – 0.88)** | **0.47 (0.24 – 0.89)** | **0.47 (0.25 – 0.89)** | **0.43 (0.22 – 0.84)** |
| Family history of hypertension | | | | | |
| No (reference) | | | | | |
| Yes | **0.51 (0.31 – 0.83)** | 0.60 (0.35 – 1.00) | **0.57 (0.34 – 0.98)** | **0.58 (0.34 – 0.99)** | 0.62 (0.36 – 1.06) |
| Self-reported medical history of diabetes | | | | | |
| No (reference) | | | | | |
| Yes | 0.85 (0.45 – 1.60) | 1.00 (0.51 – 2.00) | 1.06 (0.53 – 2.10) | 1.05 (0.53 – 2.10) | 1.09 (0.54 – 2.19) |
| BP measurement in the year before study enrollment | | | | | |
| No (reference) | | | | | |
| Yes | **0.31 (0.18 – 0.54)** | **0.48 (0.27 – 0.86)** | **0.51 (0.28 – 0.95)** | **0.52 (0.28 – 0.96)** | **0.46 (0.25 – 0.87)** |
| **Socioeconomic Factors** | | | | | |
| Monthly Family Income | | | | | |
| ≥1 000 PAB (reference) | | | | | |
| 250 – 999 PAB | 0.90 (0.45 – 1.78) | | 0.83 (0.40 – 1.74) | 0.83 (0.40 – 1.74) | 0.69 (0.33 – 1.48) |
| <250 PAB | 0.32 (0.42 – 1.70) | | 0.90 (0.42 – 1.92) | 0.88 (0.40 – 1.94) | 0.62 (0.26 – 1.50) |
| Regions | | | | | |
| Urban (reference) | | | | | |
| Non-urban | **1.41 (0.99 – 2.01)** | | | 1.07 (0.70 – 1.62) | 0.94 (0.60 – 1.47) |
| Education | | | | | |
| Higher education (reference) | | | | | |
| Secondary | 0.79 (0.43 – 1.46) | | | | 1.39 (0.74 – 2.64) |
| No/primary education | 1.19 (0.65 – 2.18) | | | | **2.27 (1.06 – 4.86)** |

SBP=systolic blood pressure. DBP=diastolic blood pressure. OR=odds ratio. CI=confidence interval. BP=blood pressure. BMI=body mass index. PAB=Panamanian Balboa.

Model 1= adjusted by sex, age group, ethnicity, BMI, physical inactivity, family history of hypertension, self-reported medical history of diabetes, tobacco consumption, and BP measurement in the year before study enrollment.

Model 2= further adjusted by monthly family income.

Model 3= further adjusted by region.

Model 4= further adjusted by education.

[1]According to World Health Organization (WHO).
