# Supplementary material for: Prevalence of hypertension and possible risk factors of hypertension unawareness among individuals aged 30–75 years from two Panamanian provinces: Results from population-based cross-sectional studies, 2010 and 2019
Source: PLoS One. 2022 Nov 28;17(11):e0276222. doi: 10.1371/journal.pone.0276222 (PMC9704556; doi:10.1371/journal.pone.0276222)
Supplement: S5 Table — Odds ratio (OR) and 95% confidence intervals (CIs). (PDF) [file pone.0276222.s005.pdf]

**S5 Table.**

**Crude and adjusted logistic regression analysis of hypertension unawareness (SBP/DBP ≥140/90 mmHg without a self-reported medical history of hypertension) in hypertensive participants aged between 30 and 75 years in the ENSPA study in the provinces of Panama and Colon. Odds ratio (OR) and 95% confidence intervals (CIs).**

| | | **Adjusted analysis OR (95% CI)** | | | |
|---|---|---|---|---|---|
| **Demographic Factors** | **Crude analysis OR (95% CI)** | Model 1 | Model 2 | Model 3 | Model 4 |
| Sex | | | | | |
| Female (reference) | | | | | |
| Male | **2.19 (1.62 – 2.97)** | **2.13 (1.42 – 3.20)** | **2.15 (1.44 – 3.22)** | **2.13 (1.42 – 3.20)** | **2.09 (1.37 – 3.17)** |
| Age | | | | | |
| ≥50 years (reference) | | | | | |
| <50 years | **2.22 (1.60 – 3.09)** | **2.29 (1.54 – 3.40)** | **2.29 (1.50 – 3.51)** | **2.24 (1.46 – 3.42)** | **2.43 (1.57 – 3.76)** |
| Ethnicity | | | | | |
| Caucasian (reference) | | | | | |
| Afro-Panamanian | 1.27 (0.77 – 2.07) | 1.66 (0.85 – 3.24) | 1.58 (0.81 – 3.10) | 1.59 (0.80 – 3.16) | 1.60 (0.80 – 3.21) |
| Mestizo | 0.89 (0.57 – 1.38) | 1.24 (0.70 – 2.18) | 1.20 (0.68 – 2.11) | 1.18 (0.66 – 2.10) | 1.18 (0.66 – 2.09) |
| Indigenous | 2.35 (0.94 – 5.85) | 1.62 (0.61 – 4.30) | 1.52 (0.57 – 4.03) | 1.32 (0.48 – 3.64) | 1.32 (0.47 – 3.71) |
| Others (including Asians) | 2.13 (0.81 – 5.64) | 1.82 (0.64 – 5.16) | 1.58 (0.56 – 4.47) | 1.62 (0.58 – 4.56) | 1.37 (0.47 – 3.99) |
| **Risk factors for hypertension** | | | | | |
| Tabaco consumption | | | | | |
| Non-smoker (reference) | | | | | |
| Ex-smoker | 0.89 (0.37 – 2.12) | 1.34 (0.38 – 4.72) | 1.25 (0.34 – 4.63) | 1.19 (0.33 – 4.25) | 1.24 (0.33 – 4.68) |
| Current smoker | 1.53 (0.68 – 3.42) | 0.94 (0.34 – 2.58) | 0.80 (0.30 – 2.16) | 0.81 (0.30 – 2.24) | 0.73 (0.27 – 1.98) |
| Body mass index[1] | | | | | |
| Healthy weight (reference) | | | | | |
| Underweight | 0.69 (0.11 – 4.49) | 0.89 (0.24 – 3.35) | 0.79 (0.21 – 2.93) | 0.83 (0.22 – 3.08) | 0.88 (0.24 – 3.32) |
| Overweight | 0.96 (0.60 – 1.54) | 1.09 (0.65 – 1.85) | 1.18 (0.70 – 1.99) | 1.18 (0.70 – 1.97) | 1.16 (0.68 – 1.98) |
| Obesity | **0.60 (0.39 – 0.94)** | 0.79 (0.47 – 1.34) | 0.80 (0.48 – 1.34) | 0.80 (0.48 – 1.34) | 0.76 (0.44 – 1.29) |
| Physical inactivity | | | | | |
| No (reference) | | | | | |
| Yes | 0.95 (0.68 – 1.33) | 0.92 (0.61 – 1.39) | 0.90 (0.59 – 1.36) | 0.91 (0.60 – 1.39) | 0.88 (0.57 – 1.36) |
| Family history of hypertension | | | | | |
| No (reference) | | | | | |
| Yes | **0.17 (0.12 – 0.24)** | **0.18 (0.12 – 0.27)** | **0.18 (0.12 – 0.27)** | **0.18 (0.12 – 0.27)** | **0.18 (0.11 – 0.27)** |
| Self-reported medical history of diabetes | | | | | |
| No (reference) | | | | | |
| Yes | **0.31 (0.18 – 0.54)** | 0.57 (0.32 – 1.02) | **0.54 (0.30 – 0.95)** | **0.55 (0.31 – 0.97)** | 0.55 (0.31 – 1.00) |
| BP measurement in the year before study enrollment | | | | | |
| No (reference) | | | | | |
| Yes | **0.25 (0.18 – 0.36)** | **0.35 (0.23 – 0.52)** | **0.35 (0.23 – 0.53)** | **0.34 (0.23 – 0.52)** | **0.35 (0.23 – 0.53)** |
| **Socioeconomic Factors** | | | | | |
| Monthly Family Income | | | | | |
| ≥1 000 PAB (reference) | | | | | |
| 250 – 999 PAB | **1.68 (1.08 – 2.65)** | | | | 1.39 (0.68 – 2.81) |
| <250 PAB | **1.69 (1.03 – 2.75)** | | | | 1.00 (0.47 – 2.10) |
| Regions | | | | | |
| Urban (reference) | | | | | |
| Non-urban | **1.78 (1.27 – 2.50)** | | | 1.48 (0.95 – 2.31) | **1.63 (1.03 – 2.60)** |
| Education | | | | | |
| Higher education (reference) | | | | | |
| Secondary | 1.49 (0.94 – 2.37) | | 1.58 (0.86 – 2.92) | 1.54 (0.84 – 2.84) | 1.42 (0.72 – 2.81) |
| No/primary education | 1.13 (0.69 – 1.85) | | 1.07 (0.56 – 2.05) | 0.98 (0.51 – 1.87) | 0.95 (0.45 – 1.99) |

SBP=systolic blood pressure. DBP=diastolic blood pressure. OR=odds ratio. CI=confidence interval. BP=blood pressure. BMI=body mass index. PAB=Panamanian Balboa.

Model 1= adjusted by sex, age group, ethnicity, BMI, physical inactivity, family history of hypertension, self-reported medical history of diabetes, tobacco consumption, and BP measurement in the year before study enrollment.

Model 2= further adjusted by education.

Model 3= further adjusted by region.

Model 4= further adjusted by monthly family income.

[1]According to World Health Organization (WHO).
