# Supplementary material for: Prevalence of hypertension and possible risk factors of hypertension unawareness among individuals aged 30–75 years from two Panamanian provinces: Results from population-based cross-sectional studies, 2010 and 2019
Source: PLoS One. 2022 Nov 28;17(11):e0276222. doi: 10.1371/journal.pone.0276222 (PMC9704556; doi:10.1371/journal.pone.0276222)
Supplement: Attachment — Submitted filename: Response to Reviewers.docx [file pone.0276222.s006.docx]

**Rebuttal letter**

**Reference: No PONE-D-21-34832**

**Revised title** “Prevalence of hypertension and possible risk factors of hypertension unawareness among individuals aged 30–75 years from two Panamanian provinces: Results from population-based cross-sectional studies, 2010 and 2019.”

Thank you for the opportunity to read the manuscript and improve it with your comments.

**Reviewers' comments:**

**Reviewer #1**

"*The paper actually does not define prevalence of hypertension and undiagnosed hypertension, and Figure 1 makes the situation more confusing. Those with hypertension are defined as having BP ≥ 140/90 mmHg or self-reported hypertension, where the denominator of the prevalence is presumed to be the entire population. For undiagnosed hypertension, the numerator is those without a self-report of a hypertension but with BP ≥ 140/90 mmHg, but the denominator of prevalence estimate is not clear (i.e., is it the entire population or just the population with a self-report of hypertension). The figure shows that approximately half of the population is excluded from the estimation of uncontrolled hypertension, largely because they are “within self-reported medical history and measured SBP/DBP < 140/90.” This suggests that the prevalence of undiagnosed hypertension is restricted to those with a self-report; however, it makes no sense at all to exclude those with controlled hypertension! That the prevalence of hypertension and undiagnosed hypertension in men is 46.5% and 52.3% suggests again that the prevalence of undiagnosed hypertension is restricted to those with a self-reported hypertension (how else could a larger proportion of participants have undiagnosed hypertension than have hypertension). That these prevalence estimates are shifting denominators is not clearly explained and will confuse many readers (perhaps even including this reviewer?).*

*In a closely related issue, the numbers in the numerator and denominator of the prevalence estimates are not provided in either the text or tables and would help the reader understand what is being calculated. Please provide the “n”. The finding of a decreased prevalence of hypertension but an increased prevalence of undiagnosed hypertension is surprising.*"

**Reply:**

Thank you for taking the time for reading the submitted manuscript. We apologize that in the previous version of the manuscript, the prevalence calculations for hypertension and for undiagnosed hypertension were not defined. To clarify this issue, we have first, changed the term "undiagnosed hypertension" to "unawareness of hypertension" throughout the revised manuscript.

In our manuscript, hypertension was defined as (a) having a self-reported medical history of hypertension or (b) having a mean SBP ≥140 and/or DBP ≥90 mmHg (regardless of their self-reported medical history of hypertension) or (c) a combination of both. This sample constituted our study population for estimating the prevalence of hypertension unawareness. Unawareness of hypertension was defined as having a mean SBP ≥140 and/or DBP ≥90 mmHg and no self-reported medical history of hypertension.

Following the Reviewer suggestions, we have now:

1. **Renamed the title** to:

"Prevalence of hypertension and possible risk factors of hypertension unawareness among individuals aged 30–75 years from two Panamanian provinces: Results from population-based cross-sectional studies, 2010 and 2019."

2. Rewrote the **Method** section between the lines 153 to 159 as follows:

"Hypertension was defined as (a) having a self-reported medical history of hypertension or (b) having a mean SBP ≥140 and/or DBP ≥90 mmHg (regardless of their self-reported medical history of hypertension) or (c) a combination of both [27]. This sample constituted our study population for estimating the prevalence of hypertension unawareness. Unawareness of hypertension was defined as having a mean SBP ≥140 and/or DBP ≥90 mmHg and no self-reported medical history of hypertension. We excluded participants who denied self-reported medical history of hypertension and were also missing the second and/or third BP measurement(s) (n=120 in the PREFREC study; n=487 in the ENSPA study) (Fig 1)."

3. Edited the abstract under the **Method** section as follows:

"Individuals aged 30–75 years who had (a) self-reported history of hypertension or (b) blood pressure (BP) ≥140/90mmHg or (c) a combination or both were classified as hypertensive. Participants with BP≥140/90mmHg who denied a history of hypertension were considered unaware of the condition."

4. Modified Figure 1; where we wrote down the weighted and unweighted number of participants with hypertension with and without self-reported medical history of hypertension.

Furthermore, as shown in Figure 2, the reader can appreciate that the numerator for the prevalence of hypertension is the weighted number of individuals with the condition among the population under study, while the prevalence of hypertension unawareness was calculated only among those individuals who had hypertension (self-reported or detected after blood pressure assessment) as it is shown in previous papers [1–4].

“*The populations in the two waves of the assessments are quite different, for example those in the ENSPA had a quite different distribution of education (with a larger number with secondary education, but fewer in either the low or high educated groups), had higher income, were more likely non-urban, and were more likely obese and physically inactive. The paper would be substantially strengthened to see if the differences in the prevalence of hypertension and undiagnosed hypertension are attributable to these differences (which can be assessed in a multivariable model including all data and estimating the odds of positive prevalence after adjustment for the other factors)*”

**Reply**

We agree with the Reviewer that this point further strengthens the manuscript. The authors initially proposed to do so. However, due to different methodologies used in PREFREC 2010 (measuring the blood pressure in the right arm) and ENSPA 2019 (measuring it in the left arm), as we mentioned in Manuscript lines 143–151, we decided not to merge both databases for analysis. According to the literature, BP readings in the right side are higher than left ones [5,6].

Instead, our approach was to perform analysis looking for possible risk factors associated with hypertension unawareness in the PREFREC (2010) and ENSPA (2019) study, separately (Table 2); and our point estimates were consistent for many of the factors analyzed. For example, we found similar points estimates for sex (men were more likely to have unawareness) and for blood pressure assessment in the year before study enrollment (protective factor in both studies); whereas age <50 years had a higher point estimate in the ENSPA (2019) study, independent of the same median age in the study populations.

Furthermore, we found that individuals with no/primary education were more likely to be unaware in the PREFREC (2010) study, but no association was found in the ENSPA (2019) study; this finding could be due to fewer individual reporting no/primary education in the ENSPA (2019) study. Interestingly, in the PREFREC (2010) study, we found decreased odds for unawareness in obese and physical inactive individuals, whereas obesity was not associated to our outcome of interest in ENSPA (2019) study. Other possible factors were discussed in more detail in the **Discussion** section.

Despite the decision not to show the merge data from both studies, for editorial purposes, we performed logistic regression models to assess the change over time of hypertension and its unawareness with the adjustments for the model A and B shown in Manuscript Table 2, using the PREFREC study as the reference. Hypertension decreased 20% between both studies with borderline significance (Model A= OR: 0.82 [95% CI: 0.64–1.04]; Model B= 0.79 [0.62–1.02]). On the other hand, hypertension unawareness increased between 70-80% during the same period (Model A= OR: 1.81 [95% CI: 1.25–2.63]; Model B= 1.70 [1.15–2.51]).

**Reviewer #2**

"*Thank you for your great data about national prevalence of hypertension. I don't understand that half of the individuals who participated in health check-ups did not have a BP assessment. It would be helpful to understand better if you add a description of Panama's health screening system.*"

**Reply:**

The authors thank you for your comment. Panama's health screening system is based on national technical-administrative standards published in 2018 [7]. According to these standards, blood pressure should be screened on any patient attending a healthcare facility aged 18 years or older. However, we found a lack of adherence to the aforementioned standards, since half of the participants stated that attended an annual check-up denied having blood pressure measures taken in the same period of time.

Following your suggestion, we added the following statement in the revised manuscript (Lines 348 and 349) to make clear the Panamanian standards on hypertension screening:

"Of note, the current national healthcare program states that BP levels should be measured in the right arm at every health check-up in patients 18 years or older."

**References:**

1. Chau K, Girerd N, Zannad F, Rossignol P, Boivin J-M. Health-related determinants of undiagnosed arterial hypertension: a population-based study. Fam Pract 2019; 36: 276–83.
2. Zhou J, Fang S. Association between Undiagnosed Hypertension and Health Factors among Middle-Aged and Elderly Chinese Population. Int J Environ Res Public Health 2019; 16: 1214.
3. Ahmed S, Tariqujjaman Md, Rahman MdA, Hasan MdZ, Hasan MdM. Inequalities in the prevalence of undiagnosed hypertension among Bangladeshi adults: evidence from a nationwide survey. Int J Equity Health 2019; 18: 33.
4. Hasan MdM, Tasnim F, Tariqujjaman Md, Ahmed S, Cleary A, Mamun A. Examining the prevalence, correlates and inequalities of undiagnosed hypertension in Nepal: a population-based cross-sectional study. BMJ Open 2020; 10: e037592.
5. Cassidy P, Jones K. A study of inter-arm blood pressure differences in primary care. J Hum Hypertens. 2001 Aug 1;15(8):519–22.
6. O’Shea J, Murphy M. Ambulatory blood pressure monitoring: which arm? J Hum Hypertens. 2000 Apr 1;14(4):227–30.
7. Panamanian Ministry of Health. Technical-Administrative Standards: Adult and Elderly Health Programs. 2018 [cited 5 May 2021]. Available from: <https://www.educacioninterprofesional.org/sites/default/files/fulltext/2018/2018_pan_norma_adulto_adulto_mayor.pdf>
