# Supplementary material for: Prevalence of hypertension and possible risk factors of hypertension unawareness among individuals aged 30–75 years from two Panamanian provinces: Results from population-based cross-sectional studies, 2010 and 2019
Source: PLoS One. 2022 Nov 28;17(11):e0276222. doi: 10.1371/journal.pone.0276222 (PMC9704556; doi:10.1371/journal.pone.0276222)
Supplement: Attachment — Submitted filename: Response to Reviewers.docx [file pone.0276222.s007.docx]

**Rebuttal letter**

**Reference: No PONE-D-21-34832R1**

**Revised title** “Prevalence of hypertension and possible risk factors of hypertension unawareness among individuals aged 30–75 years from two Panamanian provinces: Results from population-based cross-sectional studies, 2010 and 2019.”

**Reviewers' comments:**

**Reviewer #1**

*"The attempt of the authors to be responsive to the previous suggestions are appreciated, particularly the specificity in providing the definitions for hypertension and unawareness of hypertension. The authors also followed the suggestion to provide multivariable modeling for predictors of hypertension; however, unfortunately this reviewer must not have been clear in the suggestion. The lead sentence of the discussion is “… findings indicate a decline in the prevalence of hypertension between the PREFREC (2010) and 299 ENSPA (2019) studies, and an increase in the prevalence of hypertension unawareness.” The discussion follows that this may be attributable changes in exposures including increases in obesity and changes in urban/rural status. The difference could also be potentially attributed to including a lower education in ENSPA than PREFREC and much higher levels of physical inactivity. The suggestion that was apparently not clear in the previous review was to consider one multivariable model that included the data from both surveys, where the prevalence of hypertension and the prevalence of unawareness of hypertension is predicted with the period/survey as a predictor variable. This would allow the assessment of whether the odds of hypertension, and the odds of unawareness of hypertension, was different between the two surveys after adjustment for the changes in the population characteristics. While the paper is interesting as it stands, this would directly assess if there were differences in the prevalence of hypertension, and in the prevalence of unawareness of hypertension, after adjustment for differences in the individuals in the two studies."*

**Reply:**

Thank you for the time invested in revising and improving the manuscript.

Following the Reviewer's suggestions, the authors now support the claim that there were changes of hypertension prevalence and its awareness between both studies. We added multivariable logistic regression models of the prevalence of hypertension and hypertension unawareness using merged data from both studies:

In consequence, the following fragment under the statistical analysis heading was added:

"*In addition, we included in the Supporting Information (S2 and S3 Tables) crude and multivariable logistic regression models using merged data from both studies (both PREFREC and ENSPA) in which the prevalence of hypertension (S2 Table) and the prevalence of hypertension unawareness (S3 Table) were the outcome variables, respectively, with the aforementioned predictor variables together with the study period as an additional predictor (being PREFREC the reference level)."*

The following fragment was added to the **results** section:

"*As shown in the S2 and S3 Tables, after merging all the data from both studies and adjusting for all other variables, there was a 21% borderline decrease in the prevalence of hypertension (ENSPA OR: 0.79 [95% CI: 0.62–1.02], PREFREC as reference), but a 70% increase in hypertension unawareness (ENSPA OR: 1.70 [95% CI: 1.15–2.51], PREFREC as reference), respectively*."

**Reviewer #2**

*"I am very happy to be able to do this review. It's a very interesting topic and I think it's a logical conclusion overall."*

**Reply:**

The authors are grateful for your time and interest in publishing the manuscript.
