# Supplementary material for: Prevalence of hypertension and possible risk factors of hypertension unawareness among individuals aged 30–75 years from two Panamanian provinces: Results from population-based cross-sectional studies, 2010 and 2019
Source: PLoS One. 2022 Nov 28;17(11):e0276222. doi: 10.1371/journal.pone.0276222 (PMC9704556; doi:10.1371/journal.pone.0276222)
Supplement: Attachment — Submitted filename: Response to Reviewers.docx [file pone.0276222.s008.docx]

**Rebuttal letter**

**Reference: No PONE-D-21-34832R3**

**Revised title** "Prevalence of hypertension and possible risk factors of hypertension unawareness among individuals aged 30–75 years from two Panamanian provinces: Results from population-based cross-sectional studies, 2010 and 2019."

**Reviewer's comments:**

**Reviewer #1**

*"Thank you for adding the requested analysis, it does allow for the quantification of whether temporal changes are present. My only additional comment is that you note " ... there was a 21% borderline decrease in the prevalence of hypertension (ENSPA OR: 0.79 [95% CI: 0.62–1.02], PREFREC as reference), but a 70% increase in hypertension unawareness (ENSPA OR: 1.70 [95% CI: 1.15–2.51], PREFREC as reference), respectively." This is an incorrect statement, as logistic regression provides odds ratios (not relative risks), so it needs to state that the odds (not prevalence) of hypertension were 21% lower and 70% higher respectively."*

**Reply:**

We again thank you for the time invested in revising and improving the manuscript.

Following the Reviewer's suggestion, the **Results and the Discussion** sections were rewritten to reflect that logistic regression models represent changes in the *adjusted odds* between ENSPA and PREFREC studies.

Lines 251 to 255 from the **Results** section were edited as follows:

*"As shown in the S2 and S3 Tables, after merging all data from both studies and adjusting for all other variables, there was a 21% borderline decrease in the odds of having hypertension in the ENSPA (2019) study compared to the PREFREC study (ENSPA OR: 0.79; 95% CI: 0.62–1.02), but a 70% increase in the odds of being unaware (ENSPA OR: 1.70; 95% CI: 1.15–2.51), respectively."*

Lines 306 to 307 from the **Discussion** section were edited as follows:

*"Our findings indicate a borderline decrease in the odds of having hypertension between the PREFREC (2010) and ENSPA (2019) studies, but an increase in the odds of being unaware of hypertension."*

**Journal Requirements:**

The Reference list was revised at the request of the journal. All references were complete and correct, except reference 52 which has been updated, but did not alter the analysis of our discussion. Therefore, no changes have been made to the reference list.
